# Ultrasound characteristics of endometrial receptivity in patients with intrauterine adhesion following endometrial polyp surgery and their impact on pregnancy

**DOI:** 10.3389/fphys.2025.1645131

**Published:** 2025-08-08

**Authors:** Wenying Shen, Lanxiang Yan

**Affiliations:** Department of Ultrasound, Shiyan People’s Hospital, Shenzhen, Guangdong, China

**Keywords:** endometrial receptivity, endometrial polyp, intrauterine adhesion, ultrasound parameters, pregnancy outcome

## Abstract

**Objective:**

This study endeavors to investigate the ultrasound characteristics of endometrial receptivity (ER) in patients with intrauterine adhesion (IUA) following endometrial polyp surgery and their impact on pregnancy.

**Methods:**

Sixty patients with IUA following endometrial polyp surgery were selected as the observation group. Another 60 healthy women undergoing physical examinations during the same period were selected as the control group. Transvaginal three-dimensional ultrasound was used to assess ER parameters, including endometrial thickness (ET), pulsatility index (PI), resistance index (RI), endometrial volume (EV), vascularization index (VI), flow index (FI), and vascularization-flow index (VFI). Patients in the observation group underwent hysteroscopic adhesiolysis and were categorized into mild, moderate, and severe adhesion subgroups based on intraoperative findings. Ultrasound parameters were compared among subgroups. After a 2-year follow-up, patients were further divided into pregnant and non-pregnant groups according to outcomes, and their ultrasound parameters were compared. Multivariate Logistic regression analysis was performed to identify factors influencing pregnancy outcomes. ROC curve analysis was used to evaluate the predictive value of ultrasound parameters for pregnancy outcomes.

**Results:**

The observation group had a thinner ET, smaller EV, VI, FI, and VFI, and higher PI and RI in contrast to the control group (*P* < 0.05). ET was thinner, EV, VI, FI, and VFI were lower, while RI and PI were higher in the moderate and severe adhesion groups compared to the mild group. These differences were more pronounced in the severe group than in the moderate group (*P* < 0.05). The non-pregnant group had a thinner ET, smaller EV, VI, FI, VFI, PI, and RI compared to the pregnant group (*P* < 0.05). Multivariate Logistic regression analysis revealed that ET, EV, PI, VI, and FI were influential factors affecting pregnancy outcomes in the observation group. ROC curve analysis demonstrated that the AUC for predicting pregnancy in the observation group were 0.796 for ET, 0.736 for EV, 0.760 for PI, 0.752 for VI, 0.652 for FI, and 0.958 for their combined assessment (*P* < 0.05).

**Conclusion:**

ER ultrasound parameters have high evaluative value in assessing patients with IUA following endometrial polyp surgery and can effectively predict pregnancy outcomes.

## Introduction

Intrauterine adhesion (IUA), a prevalent gynecological condition, has emerged as the primary etiology of uterine-related infertility, particularly in the context of rising rates of intrauterine surgical procedures (Lin et al., 2023). IUA arises due to trauma inflicted upon the endometrial tissue (Parashar et al., 2023), frequently associated with severe clinical manifestations that significantly compromise reproductive function, including menstrual irregularities, infertility, or recurrent pregnancy loss (Lee et al., 2021). Various therapeutic strategies have been investigated to mitigate its pathological characteristics, including transcervical resection of adhesions, intrauterine contraceptive devices, anti-adhesive barriers, and periodic hormonal therapies (Lin et al., 2023; Ai et al., 2020), with the advent of hysteroscopy facilitates the evolution of more sophisticated techniques for adhesiolysis under direct endoscopic visualization (Koythong and Guan, 2020).

Since its relation to infertility, IUA may have a impact on reproductive ability for women. Infertility stands as a pervasive and challenging global medical issue. It not only severely undermines the quality of life for couples and family wellbeing but also has implications for a nation’s medical standards and reproductive health (Priskorn et al., 2021). Key pregnancy-outcome factors include embryo quality, endometrial receptivity (ER), and embryo-endometrium sync. ER is crucial, referring to the endometrial state enabling blastocyst adhesion, invasion, and implantation for successful implantation (Zajicek et al., 2022). In detail, the endometrium, which encompasses the superior inner cortex as well as the mucosa, plays a pivotal role in fertility and the maintenance of reproductive health. In clinical practice, the assessment of endometrial thickness (ET) holds significant importance for the diagnosis of disorders associated with the endometrium (Mariani et al., 2021; Long et al., 2020; Wang et al., 2022). ET is frequently utilized as an indicator to assess ER. When the endometrium is excessively thin, it creates an unfavorable environment for embryonic implantation and subsequent development (Wang et al., 2022; Zhao et al., 2014). Endometrial pathological biopsy is conventionally regarded as the gold-standard method for assessing ER. However, its clinical utility is constrained due to its invasive nature. In contrast, ultrasound is more extensively utilized for ER evaluation (Costello et al., 2019; Ni et al., 2019). Up until now, three-dimensional ultrasound has gained extensive application in clinical medicine. It is capable of generating a continuous series of anatomical images, which are highly valuable for diagnostic purposes (Wang et al., 2022).

Given that existing research predominantly focuses on therapeutic strategies for IUA, there remains a critical gap in studies specifically investigating the ultrasound characteristics of ER in patients with IUA treated by transvaginal three-dimensional ultrasound following endometrial polyp surgery. This study systematically analyzes variations in ER parameters using three-dimensional ultrasound and their correlation with pregnancy outcomes, aiming to provide objective imaging evidence for optimizing postoperative fertility management in clinical practice.

## Materials and methods

### Ethics statement

Ethical approval for the study was granted by the ethics committee of Shiyan People’s Hospital, Bao’an District, and informed consent was obtained from all participants.

### Study population

Sixty patients with IUA following endometrial polyp surgery admitted to Shiyan People’s Hospital, Bao’an District, from August 2022 to March 2023 were selected as the observation group. Another 60 healthy women (with no history of adverse pregnancy outcomes and regular menstrual cycles) who underwent physical examinations at Shiyan People’s Hospital, Bao’an District, during the same period were selected as the control group.

### Inclusion criteria

Patients who had undergone hysteroscopic resection of endometrial polyps and were subsequently diagnosed with IUA upon follow-up hysteroscopy—characterized by central adhesions with widened anterior-posterior wall fusion, partial obstruction of the uterine horns, or asymmetrical uterine cavity contours, and in some cases, small cystic cavities due to mixed adhesions. Additional criteria included a clear desire for future fertility, regular menstrual cycles, confirmed normal follicular development and ovulation, willingness to comply with follow-up protocols, no recurrence of adhesions during the follow-up period, and availability of complete clinical data.

### Exclusion criteria

Patients were excluded if they had other intrauterine pathologies (e.g., uterine fibroids), a surgical history involving the uterus or ovaries, comorbid endocrine disorders such as hyperthyroidism or polycystic ovary syndrome, congenital uterine malformations, tubal diseases, major organ dysfunction (heart, liver, or kidneys), or were diagnosed with malignant tumors.

### Classification of IUA severity

The severity of IUA was assessed via hysteroscopic examination based on the March classification criteria (March et al., 1978). Mild IUA was defined as involvement of less than one-fourth of the uterine cavity, with thin or filmy adhesions and clearly visible tubal ostia and upper uterine cavity. Moderate IUA involved one-fourth to three-fourths of the cavity, characterized by the presence of adhesions without uterine wall fusion, and partial occlusion of the tubal ostia and upper cavity. Severe IUA was defined as involvement of more than three-fourths of the uterine cavity, with thick adhesions or fusion of the uterine walls, and complete occlusion of the tubal ostia and upper cavity. According to these criteria, the 60 patients in the observation group were categorized into mild, moderate, and severe adhesion subgroups.

### Ultrasound examination method

Both groups underwent examinations on the day of ovulation using a Voluson E10 transvaginal three-dimensional ultrasound system (GE Healthcare, United States). A 3D intracavitary volume probe with a frequency of 5.0–7.5 MHz was used. Prior to the scan, participants emptied their bladders and were positioned in the lithotomy posture. After disinfection, coupling gel was applied to the probe, which was then gently inserted into the posterior vaginal fornix. Multiplanar scanning (sagittal, coronal, and transverse) of the pelvis was performed to assess the uterus, adnexa, and endometrium. ET was measured in the longitudinal plane of the uterus by identifying the maximum distance between the anterior and posterior endometrial-myometrial junctions, perpendicular to the midline of the uterine cavity. ET was measured three times, and the average was calculated. Using Doppler imaging, uterine arteries were identified at the level of the internal cervical os on each side. Once three consistent waveforms were recorded, pulsatility index (PI) and resistance index (RI) were measured, and bilateral averages were used for analysis. The 3D mode was activated to acquire volumetric and reconstructed data. The endometrial contour was outlined, and the endometrial volume (EV), vascularization index (VI), flow index (FI), and vascularization-flow index (VFI) were calculated. Image analysis was performed using VOCAL software, with each parameter measured three times and averaged. All examinations were conducted by ultrasound physicians with over 10 years of clinical experience who had undergone standardized training.

### Observation indicators

(1) Ultrasound parameters: ET, EV, PI, RI, VI, FI, and VFI were compared between the control and observation groups.
(2) Ultrasound parameters among patients with different degrees of IUA severity were compared.
(3) Patients in the observation group were followed up for 2 years after hysteroscopic adhesiolysis, with outpatient or telephone follow-up conducted every 3 months during the follow-up period. Based on pregnancy status during follow-up, patients were divided into pregnant and non-pregnant groups, and their ultrasound parameters were compared.
(4) ROC curves were plotted to analyze the clinical value of ultrasound parameters in predicting pregnancy in patients with IUA following endometrial polyp surgery, both individually and in combination.
(5) Multivariate Logistic regression analysis was implemented to identify factors influencing pregnancy outcomes in patients with IUA following endometrial polyp surgery.

### Statistical methods

All data were processed using SPSS 25.0 software (IBM SPSS Statistics, Chicago, IL, United States). Categorical data were expressed as cases and percentages (%), with comparisons between groups using the chi-square test. Measurement data were tested for normality using the Shapiro-Wilk method. Normally distributed measurement data were expressed as means ± standard deviations, with comparisons between groups using the independent samples *t*-test; while comparisons among multiple groups were performed using one-way analysis of variance (ANOVA), followed by LSD *post hoc* tests. ROC curves were plotted to analyze the predictive value of ER ultrasound parameters for pregnancy. Multivariate Logistic regression analysis was executed to identify factors influencing pregnancy. A *P*-value <0.05 was considered statistically significant.

## Results

### General information

There were no noticeable differences in age, body mass index, educational level, or age at menarche between the control and observation groups (*P* > 0.05; Table 1).

**TABLE 1:** Comparison of general information between the control group and the observation group.

Items	Control group (*n* = 60)	Observation group (*n* = 60)	*χ* ^*2*^/*t*	*P*
Age (years)	32.68 ± 3.72	33.93 ± 3.86	−1.807	0.073
BMI (kg/m^2)^	22.58 ± 1.32	22.49 ± 1.28		
Educational level			0.378	0.706
High school and below	18 (30.00)	20 (33.33)		
College degree and above	42 (70.00)	40 (66.67)		
Age at menarche (years)	14.20 ± 2.37	13.85 ± 2.07	0.860	0.390

### Ultrasound parameters among patients with varying severity of IUA

Compared with the mild group, both the moderate and severe adhesion groups showed reduced ET, EV, VI, FI, and VFI, along with elevated PI and RI. These trends were more pronounced in the severe group than in the moderate group (*P* < 0.05; Table 2).

**TABLE 2:** Ultrasound parameters among patients with varying severity of IUA.

Index	Mild adhesion group (*n* = 21)	Moderate adhesion group (*n* = 26)	Severe adhesion group (*n* = 13)	F	*P*
ET (mm)	5.31 ± 0.93	4.55 ± 0.75	3.70 ± 0.78^ab^	15.590	<0.001
EV (cm^3^)	3.14 ± 1.08	2.26 ± 0.84^b^	1.50 ± 0.77^ab^	13.369	<0.001
PI	2.59 ± 0.44	3.00 ± 0.47^b^	3.51 ± 0.65^ab^	13.514	<0.001
RI	0.75 ± 0.08	0.83 ± ±0.08^b^	0.91 ± 0.09^ab^	15.352	<0.001
VI	3.36 ± 0.63	2.87 ± 0.51^b^	2.31 ± 0.56^ab^	14.148	<0.001
FI	24.75 ± 4.31	21.16 ± 3.51^b^	17.29 ± 4.01^ab^	14.819	<0.001
VFI	0.69 ± 0.15	0.51 ± 0.14^b^	0.42 ± 0.07^ab^	22.721	<0.001

Note: ^a^ *P* < 0.05 vs. moderate adhesion group; ^b^ *P* < 0.05 vs. mild adhesion group.

### Ultrasound parameters between the observation and control groups

The observation group had a thinner ET, smaller EV, VI, FI, and VFI, and higher PI and RI in contrast to the control group (*P* < 0.05; Table 3).

**TABLE 3:** Comparison of ultrasound parameters between the control group and the observation group.

Index	Control group (*n* = 60)	Observation group (*n* = 60)	*t*	*P*
ET (mm)	8.78 ± 1.56	4.63 ± 1.00	17.338	<0.001
EV (cm^3^)	4.99 ± 2.34	2.40 ± 1.10	7.742	<0.001
PI	2.46 ± 0.52	2.97 ± 0.60	−4.948	<0.001
RI	0.78 ± 0.04	0.82 ± 0.10	−2.471	0.015
VI	4.35 ± 1.67	2.92 ± 0.68	6.146	<0.001
FI	27.80 ± 6.15	21.58 ± 4.75	6.202	<0.001
VFI	1.34 ± 0.53	0.55 ± 0.17	10.887	<0.001

### Ultrasound parameters between the pregnant and non-pregnant groups

After 2 years of follow-up, 24 out of 60 patients with IUA following endometrial polyp surgery successfully conceived, with a pregnancy success rate of 40.00%. The non-pregnant group had a thinner ET, smaller EV, VI, FI, VFI, PI, and RI compared to the pregnant group (*P* < 0.05; Table 4).

**TABLE 4:** Comparison of ultrasound parameters between the pregnant group and non-pregnant group in observation group.

Index	Non-pregnant group (*n* = 36)	Pregnant group (*n* = 24)	*t*	*P*
ET (mm)	4.23 ± 0.75	5.23 ± 1.05	−4.305	<0.001
EV (cm^3)^	2.03 ± 0.85	2.96 ± 1.20	−3.524	0.001
PI	2.76 ± 0.43	3.28 ± 0.68	−3.686	0.001
RI	0.78 ± 0.08	0.87 ± 0.11	−3.656	0.001
VI	2.70 ± 0.48	3.25 ± 0.80	−3.318	0.002
FI	20.36 ± 4.13	23.41 ± 5.10	−2.550	0.013
VFI	0.42 ± 0.03	0.75 ± 0.06	−28.161	<0.001

### Multivariate logistic regression analysis of factors influencing pregnancy

Using whether the patient conceived as the dependent variable (yes = 1, no = 0) and ET, EV, PI, RI, VI, FI, and VFI as independent variables, Logistic regression analysis identified hat ET, EV, PI, VI, and FI were significant influencing factors for pregnancy outcomes in the observation group (*P* < 0.05; Table 5).

**TABLE 5:** Multivariate Logistic regression analysis of influencing factors of pregnancy in the observation group.

Variable	β	SE	Wald	*P*	OR (95% CI)
ET (mm)	1.256	0.575	4.765	0.029	3.510 (1.137∼10.837)
EV (cm^3^)	1.213	0.514	5.571	0.018	3.365 (1.229∼9.214)
PI	1.960	0.880	4.955	0.026	7.098 (1.264∼39.864)
VI	1.843	0.652	7.984	0.005	6.315 (1.759∼22.677)
FI	0.289	0.118	5.950	0.015	1.335 (1.058∼1.683)

### Predictive value of ultrasound parameters for pregnancy

**FIGURE 1:**
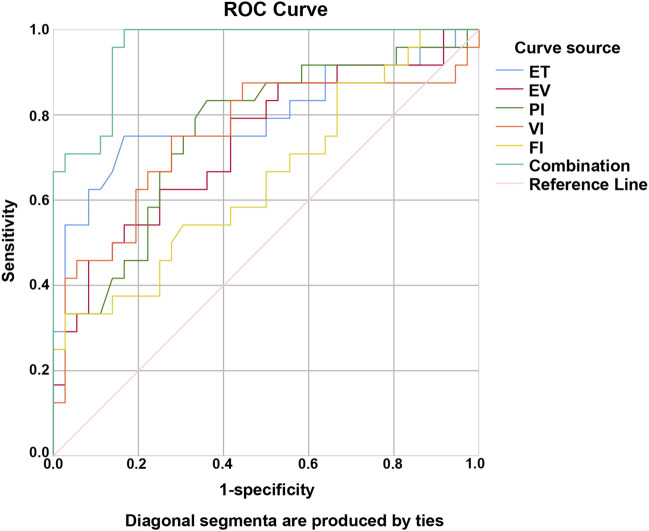
ROC curve for predicting pregnancy in the observation group using ultrasound parameters.

ROC curve analysis elucidated that AUC for predicting pregnancy in the observation group were 0.796 for ET, 0.736 for EV, 0.760 for PI, 0.752 for VI, and 0.652 for FI, while the combined assessment of these parameters yielded an AUC of 0.958, indicating the highest predictive value when used in combination (Table 6; Figure 1).

**TABLE 6:** Predictive value of ultrasound parameters on pregnancy in the observation group.

Variable	AUC	Optimal cutoff	Specificity	Sensitivity	Youden index	95% CI	*P*
ET (mm)	0.796	4.950	0.833	0.750	0.583	0.668∼0.924	<0.001
EV (cm^3^)	0.736	2.085	0.583	0.792	0.375	0.604∼0.868	0.002
PI	0.760	2.855	0.639	0.833	0.472	0.634∼0.887	0.001
VI	0.752	2.915	0.722	0.750	0.472	0.614∼0.890	0.001
FI	0.652	25.950	0.972	0.333	0.305	0.508∼0.797	0.047
Combination	0.958	—	0.833	1.000	0.833	0.915∼1.000	<0.001

## Discussion

The present study systematically evaluated the ultrasound characteristics of ER in patients with IUA following endometrial polyp surgery and their correlation with subsequent pregnancy outcomes. By comparing ultrasound-derived ER parameters with those of healthy controls and analyzing their association with subsequent pregnancy outcomes, we provide novel insights into the structural and functional alterations in the endometrium caused by IUA and their reproductive consequences.

Our findings indicate that patients with postoperative IUA exhibit significantly decreased ET and EV, along with reduced vascular indices—VI, FI, and (VFI—and elevated uterine artery impedance parameters—PI and RI. Moreover, patients with moderate and severe adhesions showed significantly reduced ET, EV, VI, FI, and VFI compared to those with mild adhesions, while RI and PI were notably increased. These changes were more pronounced in the severe group than in the moderate group, suggesting that increased adhesion severity is associated with poorer endometrial perfusion and receptivity. These findings highlight the utility of ultrasound parameters in stratifying IUA severity and guiding individualized treatment. These alterations reflect a compromised intrauterine environment, consistent with prior studies demonstrating that IUA, characterized by fibrotic adhesions within the uterine cavity, disrupt the endometrial architecture and impede angiogenesis, thereby limiting endometrial receptivity (Chen et al., 2023). As adequate vascular supply is a prerequisite for embryo implantation and early placental development, diminished blood perfusion may underlie the observed decline in reproductive potential.

Importantly, when stratified by pregnancy outcome during follow-up, patients in the pregnant subgroup demonstrated significantly more favorable ER parameters than those who failed to conceive. Specifically, they exhibited increased ET, EV, VI, FI, and VFI values, suggesting better-developed and more functional endometrial tissue with superior perfusion capacity. This aligns with previous findings showing that successful implantation is closely associated with higher subendometrial vascularity and volumetric indices (Peng et al., 2025; Wu et al., 2022). Interestingly, PI and RI were also higher in the pregnant group, potentially reflecting adaptive vascular remodeling rather than pathological resistance, highlighting the nuanced role of hemodynamics in implantation physiology.

Multivariate logistic regression further confirmed that ET, EV, PI, VI, and FI are independent predictors of pregnancy outcomes, reinforcing their clinical relevance. Notably, ROC analysis demonstrated that the combination of these parameters yielded superior predictive accuracy (AUC = 0.958) compared to any individual marker, supporting a multi-parametric assessment strategy. This finding is consistent with literature emphasizing the comprehensive nature of ER, which cannot be fully captured by a single measurement but instead requires integration of morphologic, hemodynamic, and functional dimensions (Craciunas et al., 2019).

Clinically, our results highlight the practical advantages of three-dimensional transvaginal ultrasound as a non-invasive, accessible, and reproducible tool for evaluating ER in patients with a history of endometrial pathology. Unlike invasive biopsy-based assessments, ultrasound allows real-time, cycle-specific monitoring, making it particularly suitable for fertility preservation and treatment planning (Elsokkary et al., 2019). In patients recovering from IUA, such dynamic evaluation enables timely identification of those at risk of poor reproductive outcomes, guiding tailored interventions such as hormonal modulation, intrauterine therapy, or assisted reproductive technologies.

This study has several limitations. First, it was conducted at a single center with a relatively limited sample size, which may limit the generalizability of the findings. Second, although we have provided a comparison of ultrasound parameters among IUA patients with different severity grades, the study did not include an inter-observer consistency analysis, which may introduce measurement bias. Third, the follow-up period was relatively short, and longer-term fertility outcomes after hysteroscopic treatment were not fully assessed. Future research should consider multicenter collaboration, larger sample sizes, inter-rater reliability testing, and extended follow-up to validate and expand upon these findings.

In conclusion, this study validates the clinical utility of three-dimensional ultrasound parameters in assessing ER status and predicting pregnancy outcomes in patients with IUA following endometrial polyp surgery. The integrated evaluation of structural, volumetric, and vascular ultrasound metrics provides a robust framework for optimizing reproductive counseling and therapeutic strategies in this challenging patient population. Future research should integrate advanced diagnostic modalities, such as contrast-enhanced ultrasonography or MRI, and explore molecular markers of endometrial receptivity to validate and extend our findings. Additionally, interventional trials leveraging ultrasound ER parameters to guide clinical decision-making could further strengthen causal inference.

## Data Availability

The raw data supporting the conclusions of this article will be made available by the authors, without undue reservation.
